# Venous thromboembolism in cancer patients: report of baseline data from the multicentre, prospective Cancer-VTE Registry

**DOI:** 10.1093/jjco/hyaa112

**Published:** 2020-07-27

**Authors:** Yasuo Ohashi, Masataka Ikeda, Hideo Kunitoh, Mitsuru Sasako, Takuji Okusaka, Hirofumi Mukai, Keiichi Fujiwara, Mashio Nakamura, Mari S Oba, Tetsuya Kimura, Kei Ibusuki, Masato Sakon

**Affiliations:** 1 Department of Integrated Science and Engineering for Sustainable Society, Chuo University, Tokyo, Japan; 2 Division of Lower Gastrointestinal Surgery, Hyogo College of Medicine, Nishinomiya, Japan; 3 Department of Medical Oncology, Japanese Red Cross Medical Center, Tokyo, Japan; 4 Department of Surgery, Yodogawa Christian Hospital, Osaka, Japan; 5 Department of Hepatobiliary and Pancreatic Oncology, National Cancer Center Hospital, Tokyo, Japan; 6 Division of Breast and Medical Oncology, National Cancer Center Hospital East, Kashiwa, Japan; 7 Department of Gynecologic Oncology, Saitama Medical University International Medical Center, Hidaka, Japan; 8 Department of Internal Medicine, Pediatrics and Cardiology, Nakamura Medical Clinic, Kuwana, Japan; 9 Department of Medical Statistics, Faculty of Medicine, Toho University, Tokyo, Japan; 10 Medical Science Department, Daiichi Sankyo Co. Ltd., Tokyo, Japan; 11 Department of Gastrointestinal Surgery, Osaka International Cancer Institute, Osaka, Japan

**Keywords:** venous thromboembolism, thrombosis, pulmonary embolism, registry, cancer

## Abstract

**Background:**

The Cancer-VTE Registry evaluates the occurrence and management of venous thromboembolism in Japanese participants with major solid tumors. Using Registry data, we evaluated the frequency of concurrent venous thromboembolism in cancer patients prior to treatment initiation by cancer type.

**Methods:**

The Cancer-VTE Registry is an ongoing (March 2017–September 2020) prospective cohort study using a nationwide, multicentre clinical registry. Participants aged ≥20 years with colorectal, lung, stomach, pancreatic, breast or gynecologic cancer, confirmed staging, ≥6 months life expectancy post-registration and who had undergone venous thromboembolism screening were managed with routine clinical care. Venous thromboembolism frequency at registration was evaluated.

**Results:**

Of 9735 participants, 571 (5.9%) had venous thromboembolism at baseline, including asymptomatic [5.5% (*n* = 540)] and symptomatic venous thromboembolism [0.3% (*n* = 31)]. Most participants with venous thromboembolism (*n* = 506, 5.2%) had deep vein thrombosis only; 65 (0.7%) had pulmonary embolism with/without deep vein thrombosis. The prevalence of distal and proximal deep vein thrombosis was 4.8% (*n* = 466) and 0.9% (*n* = 83), respectively. The highest prevalence of venous thromboembolism was for pancreatic cancer (8.5%) and the lowest for breast cancer (2.0%). Venous thromboembolism prevalence increased as cancer stage advanced.

**Conclusions:**

Although there was a marked difference in venous thromboembolism by cancer type, the data suggest that cancer stage is an important risk factor for venous thromboembolism. Thus, metastasis seems a critical risk factor for venous thromboembolism. This is the first demonstration of venous thromboembolism prevalence and risk factors in Japanese cancer patients prior to treatment.

**Trial registration:**

UMIN000024942.

## Teaser

Cancer-VTE Registry data showed that 571/9735 participants (5.9%) had venous thromboembolism at baseline. Thromboembolism prevalence was highest for pancreatic cancer and lowest for breast cancer and increased as cancer stage advanced.

## Introduction

Venous thromboembolism (VTE) comprises pulmonary embolism (PE) and deep vein thrombosis (DVT). VTE is associated with a considerable disease burden, including long-term complications and significant morbidity (1–3). Notably, among patients with cancer, the risk of VTE is estimated to be four- to seven-fold that of the general population (4–6). Furthermore, in cancer patients, VTE is associated with worse prognosis (7–10) and increased medical costs (11,12). Among 4466 cancer patients in the United States, VTE was observed to be the second most common cause of death (8).

There are several pathways that can result in an increased risk of VTE in cancer patients (13), including increased blood coagulability resulting from the release of inflammatory cytokines from cancer cells (14–16). The risk of developing VTE during cancer treatment may depend on patient-related (including age, body mass index, performance status, smoking and concomitant medical comorbidities), tumor-related (cancer type and stage) and treatment-related factors (such as surgery, use of chemotherapy, hormone therapy or immune-checkpoint inhibitor and placement of a venous catheter) (17,18). Although the association between cancer and VTE is well recognized, information on Japanese or Asian patients with cancer and VTE is currently scarce. No data from large-scale, reliable and prospective studies evaluating the frequency (prevalence and/or incidence) of VTE and treatment status have been reported in this population. Thus, the relative VTE frequencies according to different types of cancer and risk factors are not yet understood.

The Cancer-VTE Registry aimed to determine the prevalence of VTE at treatment initiation and the cumulative incidence of VTE after 1 year in patients according to cancer type (colorectal, lung, stomach, pancreatic, breast or gynecologic), to investigate risk factors associated with VTE manifestation and to clarify the current treatment landscape of VTE in cancer patients, as well as survival status. The study is ongoing. Here, we address the first of these objectives by evaluating baseline data and the prevalence of VTE prior to treatment initiation in participants enrolled in the Cancer-VTE Registry.

## Patients and methods

### Study design

The full details of the Cancer-VTE Registry design have been reported previously (19). In brief, this is a prospective cohort study based on a nationwide, multicentre clinical registry (Supplementary Fig. S1).

As far as possible, all eligible patients were consecutively registered. Registered patients were managed with routine clinical care, and no interventions were specified.

The study was initiated in March 2017 and will be completed in September 2020. Enrollment ended in January 2019, and the data discussed herein are derived from the baseline demographic and clinicopathologic information submitted at registration. For gynecologic cancers, participants were separately enrolled in an investigator-initiated study with an intervention; the data from that study were added to those of the main registry in an integrated analysis. The data cut-off for this analysis was 9 August 2019 for colorectal, lung, stomach, pancreatic and breast cancers and 30 May 2019 for gynecologic cancers.

### Ethics

The study was conducted according to the Ethical Guidelines for Medical and Health Research Involving Human Subjects and the ethical principles originating from the Declaration of Helsinki. The protocol, amendments and participant consent forms were approved by the institutional review board/independent ethics committee at each site prior to study commencement. All participants provided written informed consent at the time of registration. All participant information has been anonymized to ensure privacy.

### Participants

Enrollment details for the Cancer-VTE Registry have been published (19). All patients were registered before initiating planned cancer treatment (including chemotherapy, radiation therapy or surgery). Eligibility criteria included age ≥20 years and a diagnosis of colorectal, lung, stomach, pancreatic, breast or gynecologic cancer (comprising endometrial, cervical, ovarian, fallopian tube, and peritoneal tumors). Patients with recurrent cancer (defined as patients for whom all planned cancer treatments had been completed and who had at least 6 months of stable disease before disease progression was detected), who had previously received treatment (including chemotherapy, radiation therapy or surgery) for the primary cancer, were also eligible for registration; cases of recurrence were handled as stage IV cases. Confirmation of stage II–IV cancer with planned initiation of cancer therapy was necessary for participation (stages I–IV for gynecological cancers and stages IB–IV for lung cancer).

Participants could be outpatients or hospitalized, with an Eastern Cooperative Oncology Group Performance Status (ECOG PS) of 0, 1 or 2 (for pancreatic cancer, a PS of 0 or 1 only was permitted), with a life expectancy of ≥6 months after registration and had undergone VTE screening [lower extremity venous ultrasonography or computed tomography (CT) angiography] in the 2 months prior to registration. Venous ultrasonography of the lower extremity was standardized with the aid of the Japan Society of Ultrasonics in Medicine guidelines (20). However, VTE screening was not required if patients had a D-dimer concentration of ≤1.2 μg/ml after cancer diagnosis (regarded as non-VTE) (21). When PE was suspected due to subjective or other findings, its presence or absence was confirmed by conducting diagnostic imaging tests (such as contrast CT) at the physician’s discretion.

**Figure 1.**
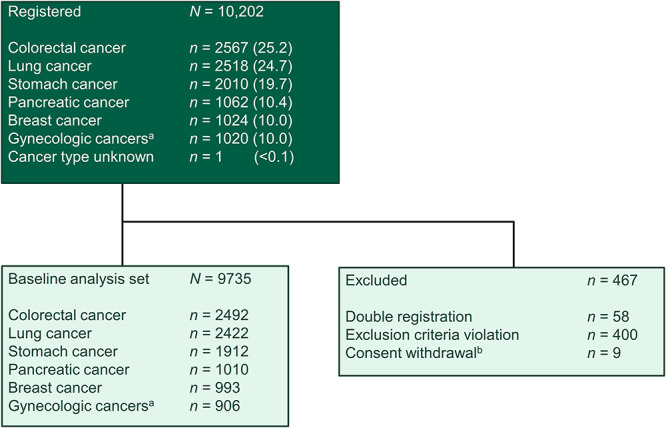
Participant flow. ^a^Includes endometrial, cervical, ovarian, fallopian tube and peritoneal cancers. ^b^No data available.

Patients with active double cancer were excluded, although multiple intramucosal cancers in one organ could be registered. There were no other specific exclusion criteria, although the investigator could exclude patients if their participation was considered inappropriate owing to compliance or safety reasons.

### Endpoints

The overall outcome measures for the study have been defined previously (19).

This article will present data derived from baseline participant registration information, including baseline demographic and clinical characteristics and an assessment of the prevalence of VTE [including symptomatic/incidental PE, and symptomatic/asymptomatic DVT (proximal and distal)] among participants with six different cancer types.

The definitions of PE and DVT have been described (19); details are also provided in Supplementary Table S1.

### Statistical analyses

The planned sample size, which was based on estimated cancer incidences among the Japanese population (22), included a total of 10 000 participants (colorectal cancer: 2500; lung cancer: 2500; stomach cancer: 2000; pancreatic cancer: 1000; breast cancer: 1000; and gynecologic cancers: 1000). Baseline demographic and clinicopathologic characteristics were reported using descriptive statistics (frequency, mean and standard deviation). All analyses (including data access, extraction and management) were performed using SAS software version 9.4 (SAS Institute Inc., Cary, NC, USA) by an external company (Mediscience Planning Inc., Tokyo, Japan).

## Results

### Participants

Between March 2017 and February 2019, 10 202 participants were registered at 162 sites in Japan, achieving both the overall target of 10 000 participants and the targets for each cancer type. At the time of registration, before the start of cancer treatment, VTE was confirmed by imaging examinations, and the VTE status at baseline was clarified. Herein, we report the results based on confirmed baseline data, which included a total of 9735 participants. The study flow is shown in Fig. 1.

Baseline demographic and clinical characteristics are shown in Table 1. The proportion of males was 51.4%, the mean age was 66.7 years and the mean body mass index was 22.6 kg/m^2^. Most participants (95.5%) had a primary cancer, while just 4.5% had cancer recurrence. The ECOG PS was 0 in 74.3% of participants. Regarding cancer stage, 35.2% had stage II, 29.8% had stage III and 24.0% had stage IV cancer. Around half of the participants had lymph node metastasis (54.5%), but only a quarter (23.0%) had distant metastasis. The most frequently observed complications were hypertension (39.0%) and diabetes (18.9%), and 18.9% had a history of organ resection.

**Table 1:** Baseline clinical characteristics

Characteristic	Baseline analysis set *N* = 9735
Sex, male	5001 (51.4)
Age, years, mean (SD)	66.7 (11.9)
≥65 years	6246 (64.2)
Weight, kg, mean (SD)	58.1 (12.0)
BMI, kg/m^2^, mean (SD)	22.6 (3.9)
Cancer occurrence	
Primary	9300 (95.5)
Recurrence	435 (4.5)
ECOG PS	
0	7235 (74.3)
1	2164 (22.2)
2	336 (3.5)
Cancer stage	
I^a^	615 (6.3)
IB^b^	460 (4.7)
II	3425 (35.2)
III	2902 (29.8)
IV	2333 (24.0)
Presence of lymph node metastasis	5309 (54.5)
Presence of distant metastasis	2238 (23.0)
Complications/comorbidities	
Hypertension	3800 (39.0)
Diabetes	1837 (18.9)
Ischemic heart disease	459 (4.7)
Atrial fibrillation	319 (3.3)
Liver dysfunction	280 (2.9)
Peptic ulcer	281 (2.9)
Heart failure	42 (0.4)
VTE risk factors	
Surgery	391 (4.0)
General anesthesia	340 (3.5)
Central vein port placement	211 (2.2)
Steroid use	184 (1.9)
Central venous catheterization	154 (1.6)
Bed rest for 4 days or more	118 (1.2)
Current smoker	1308 (13.4)
Medical history	
Organ resection	1844 (18.9)
Cerebral infarction	397 (4.1)
Gastrointestinal bleeding	175 (1.8)
Intracranial hemorrhage	121 (1.2)
VTE	80 (0.8)
TIA	49 (0.5)
Myocardial infarction	11 (0.1)

Data are shown as *n* (%) unless otherwise stated. SD, standard deviation; BMI, body mass index; ECOG PS, Eastern Cooperative Oncology Group performance status; VTE, venous thromboembolism; TIA, transient ischemic attack.

^a^Gynecologic cancers only.

^b^Lung cancer only.

### Baseline prevalence of VTE

The baseline prevalence of VTE is shown in Table 2. Overall, 571/9735 (5.9%) participants were found to have VTE at baseline. Most participants (*n* = 506, 5.2%) had DVT alone, and 65 (0.7%) had PE with or without DVT. The prevalence of asymptomatic and symptomatic VTE was 540 (5.5%) and 31 (0.3%), respectively. Of the 549 participants with DVT, 466 (4.8%) had distal and 83 (0.9%) had proximal DVT.

**Table 2:** Summary of VTE

	Baseline analysis set *N* = 9735
VTE prevalence	
All VTE	571 (5.9)
PE with/without DVT	65 (0.7)
DVT only	506 (5.2)
Type of VTE	
Symptomatic	31 (0.3)
Asymptomatic	540 (5.5)
Site of DVT	
Proximal	83 (0.9)
Distal	466 (4.8)

Data are shown as *n* (%). PE, pulmonary embolism; DVT, deep vein thrombosis.

### Analysis of VTE prevalence by cancer type and stage

**Figure 2.**
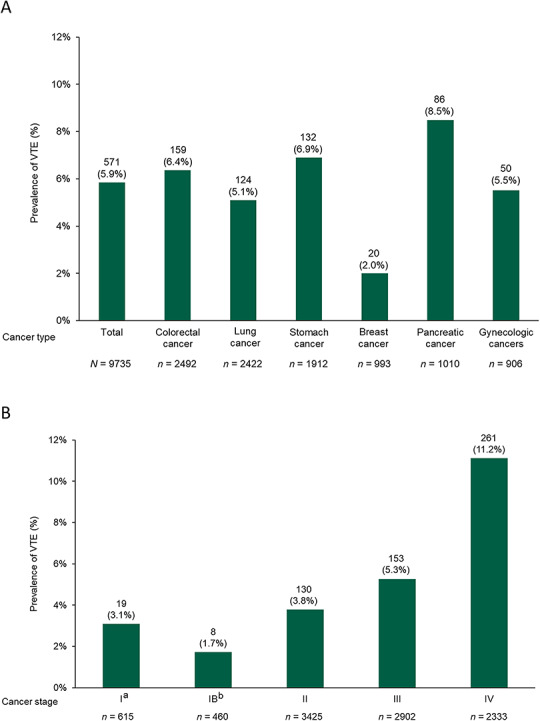
VTE prevalence by (A) cancer type and (B) cancer stage. ^a^Gynecologic cancer only. ^b^Lung cancer only. VTE, venous thromboembolism.

Figure 2A and B shows the VTE prevalence by cancer type and stage. The numbers of patients with PE (with or without DVT) and DVT and those with symptomatic or asymptomatic VTE are also described in Supplementary Table S2. The highest VTE prevalence was found in pancreatic cancer (8.5%) and the lowest in breast cancer (2.0%); the prevalence in other types of cancer was similar (5.1–6.9%). VTE prevalence appeared to increase as the cancer stage increased, reaching a level of 11.2% at stage IV.

**Figure 3.**
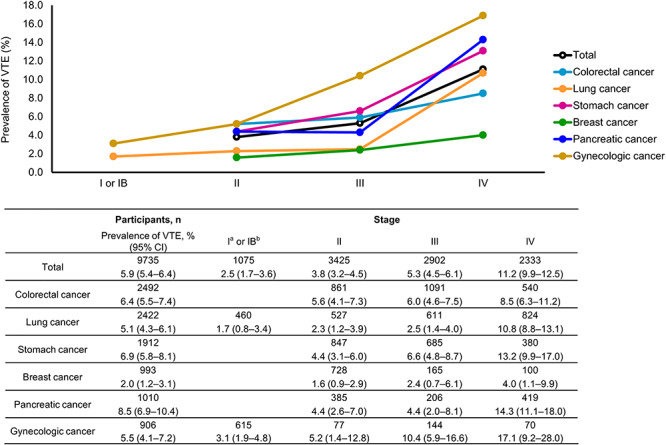
VTE prevalence by cancer type and stage. ^a^Gynecologic cancers only. ^b^Lung cancer only. Data in the table show the exact probability based on binomial distribution. CI, confidence interval.

Further, the prevalence of VTE increased rapidly for patients with advanced stomach cancer, lung cancer and pancreatic cancer (i.e. stage III or higher stages) (Fig. 3). The prevalence of VTE with confidence intervals by cancer type and stage is also shown in Fig. 3.

The regression coefficients and odds ratios of the logistic regression model obtained as a result of variable selection are shown in Supplementary Table S3. It was confirmed that female sex and higher age were independent risk factors, in addition to the combination of cancer type and stage. Applying variable selection (backward elimination) to assess the influence of other background factors on VTE and using the Akaike Information Criterion (AIC), sex, age, metastasis, history of VTE, bed rest for ≥4 days and D-dimer level were confirmed to be independent risk factors. The C-index was 0.898. The Hosmer–Lemeshow test, which was 5.81 (8 degrees of freedom; *P* = 0.67), indicated a good fit.

## Discussion

It has long been recognized that cancer patients have a high frequency (prevalence and/or incidence) of VTE, resulting in worse prognosis and high morbidity levels (7–10). However, data on the frequency and risk factors for VTE in Japanese cancer patients are currently lacking. The Cancer-VTE Registry was initiated to evaluate the frequency and management of VTE in Japanese participants with a range of solid tumors (19). The baseline data from this registry, which included 9735 participants with six cancer types, indicated that the prevalence of VTE prior to the initiation of cancer treatment was 5.9% overall. Notably, most of the cases of VTE detected at baseline were peripheral DVT; the clinical impact of DVT in these patients will be revealed after follow-up. Moreover, the frequency of VTE observed in this analysis should be interpreted and compared with caution. To put this number into the context of previous research, it is necessary to consider several possible variables, including whether the frequency was calculated during VTE screening, how the flow of VTE diagnosis was applied and the differences in background characteristics of the target patients before treatment. Asymptomatic VTE with a D-dimer ≤1.2 μg/ml may be underestimated, and asymptomatic PE without thrombosis in the lower leg may not be detectable.

The prevalence of VTE varied considerably according to the type of cancer, with the highest rates of VTE observed in pancreatic cancer, followed by stomach, colorectal, gynecologic and lung cancers. When the cancer stage was considered, VTE prevalence was found to increase with stage. It was also observed that the prevalence of VTE increased sharply in patients with stage IV stomach cancer, lung cancer and pancreatic cancer. These results are in line with data from analyses in cancer patients of other ethnicities, in which the frequency of VTE was also found to be highest in pancreatic cancer (23–26) and in tumors at a higher stage (23,25,27). Furthermore, the analysis results of risk factors affecting VTE prevalence showed that cancer stage was the dominant factor, rather than cancer type. The prevalence of VTE in gynecological cancers seemed different when viewed only by cancer type (Fig. 2A) and by cancer stage (Fig. 3). In fact, the prevalence of VTE among patients with gynecological cancer was the highest at every stage compared with other cancer types. There was a large proportion of stage I gynecologic cancers in this study, which may explain these findings. Other reasons may be that gynecologic cancers often present peritoneal metastasis, and tumors tend to be large in size (28). Thus, from the present results, it can be inferred that metastasis (i.e. advanced cancer stage) is a critical risk factor of VTE. The high prevalence of VTE in patients with pancreatic cancer may be attributable to the fact that this cancer type is commonly detected at advanced stages. In addition, mucin expression during pancreatic cancer progression may be associated, in part, with higher VTE risk (29).

The major strength of this study is the large number of patients included from real-world clinical practice, which allows the data to be extrapolated across the spectrum of Japanese cancer patients. This study has several limitations. First, only six cancer types were examined in this registry, and there are notable differences in baseline VTE risk between these six cancers; thus, further studies will be needed to expand the evidence-base and provide risk predictions for other cancer types. Second, because the registry was conducted within usual clinical practice, VTE screening was not carried out for all patients, and the screening methods used for PE were not pre-specified compared with those used for DVT. In accordance with local insurance regulations, the 5569 patients with D-dimer ≤1.2 μg/ml were not routinely screened for VTE; however, 694 of these patients underwent lower extremity venous ultrasonography or CT angiography. Of these, 33 participants were diagnosed with DVT. The prevalence of concurrent VTE in cancer patients may, therefore, be higher than the 5.9% recorded in this study.

As cancer progresses, patients undergo treatment, and follow-up is necessary. Herein, we report the prevalence of VTE prior to the initiation of cancer treatment, and subsequent publications will provide details of the course of the incidence of VTE during cancer treatment, information on VTE treatment and other cancer therapies (i.e. chemotherapy, radiotherapy and surgery) and overall survival rates. Moreover, in the future, we will examine patient background characteristics and analyze potential risk factors to further expand our understanding of VTE occurrence in cancer patients. It is expected that these data can be used to create a VTE risk score by examining the relationship between the frequency of VTE and potential VTE risk factors in cancer patients, thereby informing treatment decisions for physicians and improving outcomes for patients. We expect that further analysis of the obtained data will yield valuable information on VTE and cancer. As the data on the subject are limited, the forthcoming information that will result from these analyses is eagerly anticipated.

## Conclusions

For the first time in Japan, the prevalence of VTE in cancer patients prior to treatment initiation has been demonstrated. There was a marked difference in the VTE prevalence between the various cancer types. The present data suggest that cancer stage is the dominant risk factor for VTE rather than cancer type. From this, we inferred that metastasis, a key characteristic of advanced cancer stage, is an important risk factor for VTE.

## Funding

The Cancer-VTE registry was supported by Daiichi Sankyo Co., Ltd. The investigator-initiated study of gynecologic cancers was funded by Daiichi Sankyo Co., Ltd.

## Conflict of interest statement

Yasuo Ohashi, Masataka Ikeda, Hideo Kunitoh, Mitsuru Sasako, Takuji Okusaka, Keiichi Fujiwara, Hirofumi Mukai, Mashio Nakamura, Mari S. Oba and Masato Sakon received personal fees from Daiichi Sankyo. Tetsuya Kimura and Kei Ibusuki are employees of Daiichi Sankyo. Outside of the submitted work, the authors report the following conflicts of interest: Yasuo Ohashi reports personal fees from Sanofi; Chugai Pharmaceutical; and Shionogi. Masataka Ikeda reports personal fees from Daiichi Sankyo; Bayer Yakuhin; Kaken Pharmaceutical; Pfizer; Chugai Pharmaceutical; Taiho Pharmaceutical; and Merck Serono. Hideo Kunitoh reports personal fees from AstraZeneca; Johnson & Johnson; Taiho Pharmaceutical; Chugai Pharmaceutical; Boehringer Ingelheim; Daiichi Sankyo; Mochida Pharmaceutical; MSD; Eisai; Pfizer; EA Pharma; Covidien; Takeda Pharmaceutical; Hisamitsu Pharmaceutical; and Astellas Pharma. Takuji Okusaka reports research participation for Eisai; Novartis Pharma; Eli Lilly; Dainippon Sumitomo Pharma; AstraZeneca; Chugai Pharmaceutical; Bristol-Myers; and Taiho Pharmaceutical, and advisory board participation for Teijin Pharma; Eli Lilly; Taiho Pharmaceutical; Takeda Pharmaceutical; Eisai; Novartis Pharma; and Bristol-Myers. Hirofumi Mukai reports grant support from Kaken; Shionogi; Chugai Pharmaceutical; Immunogen; Oncotherapy; and Regenerone. Keiichi Fujiwara reports personal fees from Nihon Kayaku and Kyowa Hakko Kirin, and grants and personal fees from AstraZeneca; Pfizer; Eisai; MSD; Taiho Pharmaceutical; Zeria; Ono; GlaxoSmithKline; and Eli Lilly. Hirofumi Mukai reports grant support from the Japanese Government; Nippon Kayaku; and Eisai, personal fees from AstraZeneca; Taiho Pharmaceutical; and Takeda Pharmaceutical, and grants and personal fees from Daiichi Sankyo and Pfizer.

## Supplementary Material

Cancer-VTE_Registry_Dr_Ohashi_Revised_Supplementary_20200605_hyaa112

Cancer-VTE_Registry_Dr_Ohashi_Revised_Supplementary_List_20200605_hyaa112
