# Corrigendum to: Venous thromboembolism in cancer patients: report of baseline data from the multicentre, prospective Cancer-VTE Registry

**DOI:** 10.1093/jjco/hyaa160

**Published:** 2020-08-11

**Authors:** Yasuo Ohashi, Masataka Ikeda, Hideo Kunitoh, Mitsuru Sasako, Takuji Okusaka, Hirofumi Mukai, Keiichi Fujiwara, Mashio Nakamura, Mari S Oba, Tetsuya Kimura, Kei Ibusuki, Masato Sakon

**Affiliations:** 1 Department of Integrated Science and Engineering for Sustainable Society, Chuo University, Tokyo, Japan; 2 Division of Lower Gastrointestinal Surgery, Hyogo College of Medicine, Nishinomiya, Japan; 3 Department of Medical Oncology, Japanese Red Cross Medical Center, Tokyo, Japan; 4 Department of Surgery, Yodogawa Christian Hospital, Osaka, Japan; 5 Department of Hepatobiliary and Pancreatic Oncology, National Cancer Center Hospital, Tokyo, Japan; 6 Division of Breast and Medical Oncology, National Cancer Center Hospital East, Kashiwa, Japan; 7 Department of Gynecologic Oncology, Saitama Medical University International Medical Center, Hidaka, Japan; 8 Department of Internal Medicine, Pediatrics and Cardiology, Nakamura Medical Clinic, Kuwana, Japan; 9 Department of Medical Statistics, Faculty of Medicine, Toho University, Tokyo, Japan; 10 Medical Science Department, Daiichi Sankyo Co. Ltd., Tokyo, Japan; 11 Department of Gastrointestinal Surgery, Osaka International Cancer Institute, Osaka, Japan

The original Figure 2 published in this article mistakenly contained a duplicate of Figure 2A in the place of Figure 2B. This has now been corrected and the correct Figure 2B inserted.

