# Supplementary material for: Venous thromboembolism in cancer patients: report of baseline data from the multicentre, prospective Cancer-VTE Registry
Source: Jpn J Clin Oncol. 2020 Jul 27;50(11):1246–53. doi: 10.1093/jjco/hyaa112 (PMC7579341; doi:10.1093/jjco/hyaa112)
Supplement: Cancer-VTE_Registry_Dr_Ohashi_Revised_Supplementary_20200605_hyaa112 [file cancer-vte_registry_dr_ohashi_revised_supplementary_20200605_hyaa112.doc]

**Supplementary Data**

**Supplementary Table S1.** Definitions Used in Venous Thromboembolism Screening

| **Type of VTE** | **Criteria** |
| --- | --- |
| PE | - More than one of the following criteria must be met:  1. A new filling defect shown on CT pulmonary angiography in part of the segmental branch artery or the proximal blood vessel. 2. A new filling defect, expansion of the existing tube defect or sudden blood flow disruptions (cut-off >2.5 mm diameter) in the blood vessel shown on pulmonary angiography. 3. A new segmental perfusion defect with normal ventilation shown in ≥75% of pulmonary ventilation/perfusion scintigraphy.  - A suspected PE will be confirmed by imaging tests (e.g. CT). |
| DVT | - DVT is diagnosed when blood clots are revealed by the shadow of the thrombus in the deep vein or non-compressed abnormalities in veins, and by indirect observations as perfusion defects in venous ultrasonography. - DVT is also diagnosed in cases where blood clots are shown in deep veins by filling defects on CT angiography of the lower extremity. - Proximal DVT is defined as DVT occurring in the inferior vena cava, common iliac vein, external iliac vein, internal iliac vein, common femoral vein, femoral vein, deep femoral vein or popliteal vein. - Distal DVT is defined as DVT occurring in the peroneal, anterior tibial, posterior tibial, soleal, or sural/gastrocnemius vein. |

CT, computed tomography; DVT, deep vein thrombosis; PE, pulmonary embolism; VTE, venous thromboembolism.

**Supplementary Table S2.** Prevalence of VTE by Cancer Types and Stages

|  | **All patients, N (%)^a^** | **Prevalence of VTE, n (%)** | | | **Prevalence of PE, n (%)^b^** | | | **Prevalence of DVT, n (%)^b^** | | |
| --- | --- | --- | --- | --- | --- | --- | --- | --- | --- | --- |
|  |  | **All VTE** | **Symptomatic VTE** | **Asymptomatic VTE** | **All PE** | **Symptomatic PE** | **Asymptomatic PE** | **All DVT** | **Symptomatic DVT** | **AsymptomaticDVT** |
| Total | 9735 (100.0) | 571 (5.9) | 31 (0.3) | 540 (5.5) | 65 (0.7) | 7 (0.1) | 58 (0.6) | 549 (5.6) | 27 (0.3) | 522 (5.4) |
| **Cancer type** | | | | | | | | | | |
| Colorectal | 2492 (25.6) | 159 (6.4) | 6 (0.2) | 153 (6.1) | 13 (0.5) | 1 (0.0) | 12 (0.5) | 156 (6.3) | 5 (0.2) | 151 (6.1) |
| Lung | 2422 (24.9) | 124 (5.1) | 6 (0.2) | 118 (4.9) | 16 (0.7) | 3 (0.1) | 13 (0.5) | 118 (4.9) | 4 (0.2) | 114 (4.7) |
| Stomach | 1912 (19.6) | 132 (6.9) | 5 (0.3) | 127 (6.6) | 10 (0.5) | 1 (0.1) | 9 (0.5) | 128 (6.7) | 4 (0.2) | 124 (6.5) |
| Breast | 993 (10.2) | 20 (2.0) | 0 (0.0) | 20 (2.0) | 0 (0.0) | 0 (0.0) | 0 (0.0) | 20 (2.0) | 0 (0.0) | 20 (2.0) |
| Pancreatic | 1010 (10.4) | 86 (8.5) | 7 (0.7) | 79 (7.8) | 10 (1.0) | 0 (0.0) | 10 (1.0) | 84 (8.3) | 7 (0.7) | 77 (7.6) |
| Gynecologic | 906 (9.3) | 50 (5.5) | 7 (0.8) | 43 (4.7) | 16 (1.8) | 2 (0.2) | 14 (1.5) | 43 (4.7) | 7 (0.8) | 36 (4.0) |
| **Cancer stage** | | | | | | | | | | |
| I^c^ | 615 (6.3) | 19 (3.1) | 3 (0.5) | 16 (2.6) | 4 (0.7) | 1 (0.2) | 3 (0.5) | 18 (2.9) | 3 (0.5) | 15 (2.4) |
| IB^d^ | 460 (4.7) | 8 (1.7) | 0 (0.0) | 8 (1.7) | 2 (0.4) | 0 (0.0) | 2 (0.4) | 8 (1.7) | 0 (0.0) | 8 (1.7) |
| II | 3425 (35.2) | 130 (3.8) | 2 (0.1) | 128 (3.7) | 7 (0.2) | 1 (0.0) | 6 (0.2) | 125 (3.6) | 1 (0.0) | 124 (3.6) |
| III | 2902 (29.8) | 153 (5.3) | 4 (0.1) | 149 (5.1) | 19 (0.7) | 1 (0.0) | 18 (0.6) | 147 (5.1) | 4 (0.1) | 143 (4.9) |
| IV | 2333 (24.0) | 261 (11.2) | 22 (0.9) | 239 (10.2) | 33 (1.4) | 4 (0.2) | 29 (1.2) | 251 (10.8) | 19 (0.8) | 232 (9.9) |

^a^ This column shows the total number of patients, the number of patients with each cancer type and stage, and the percentages across all patients (N=9735).

^b^ Because there were patients who had both PE and DVT, the number of All VTE does not equal the sum of PE and DVT, as there was some patient overlap.

^c^ Gynecologic cancers only.

^d^ Lung cancer only.

DVT, deep vein thrombosis; PE, pulmonary embolism; VTE, venous thromboembolism.

# Supplementary Table S3. Risk Factors Affecting VTE Prevalence (VTE Combined Additive Model [All Data] Including Explanatory Variables)

|  | **Risk factor** | | | | |
| --- | --- | --- | --- | --- | --- |
|  |  | **Odds ratio** | | **β** | ***P* value** |
|  |  | **Estimate** | **95% CI** |  |  |
| Intercept |  | - | - | −3.37 | <.001 |
| D-dimer concentration | >1.2 μg/mL vs ≤1.2 μg/mL | 44.29 | 30.73–63.84 | 3.79 | <.001 |
| D-dimer data availability | Measured vs missing | 0.05 | 0.04–0.08 | −2.95 | <.001 |
| Sex | Female vs male | 2.31 | 1.87–2.84 | 0.84 | <.001 |
| Age | ≥65 years vs <65 years | 2.04 | 1.58–2.63 | 0.71 | <.001 |
| Cancer type | Lung cancer vs colorectal cancer | 0.76 | 0.57–1.01 | −0.28 | .06 |
|  | Gynecologic vs colorectal cancer | 0.90 | 0.61–1.34 | −0.10 | .61 |
|  | Pancreatic vs colorectal cancer | 1.12 | 0.81–1.55 | 0.11 | .50 |
|  | Breast vs colorectal cancer | 0.54 | 0.32–0.93 | −0.61 | .03 |
|  | Stomach vs colorectal cancer | 1.03 | 0.78–1.35 | 0.03 | .84 |
| Modified N classification (lymph node metastasis) | Yes vs no | 1.04 | 0.84–1.29 | 0.04 | .71 |
| Modified M classification (distant metastasis) | Metastasis vs local | 1.58 | 1.27–1.95 | 0.46 | <.001 |
| History of VTE | Yes vs no | 25.07 | 13.37–47.01 | 3.22 | <.001 |
| Bed rest for ≥4 days | Yes vs no | 2.53 | 1.50–4.25 | 0.93 | <.001 |

Hosmer–Lemeshow (HL) goodness-of-fit test: χ^2^_HL_ = 5.81; degrees of freedom = 8, *P* = .67.

C-index = 0.898.

In each comparison, the second variable is the reference value.

CI, confidence interval; VTE, venous thromboembolism.

**Supplementary Figure S1**. Cancer-VTE Registry Design


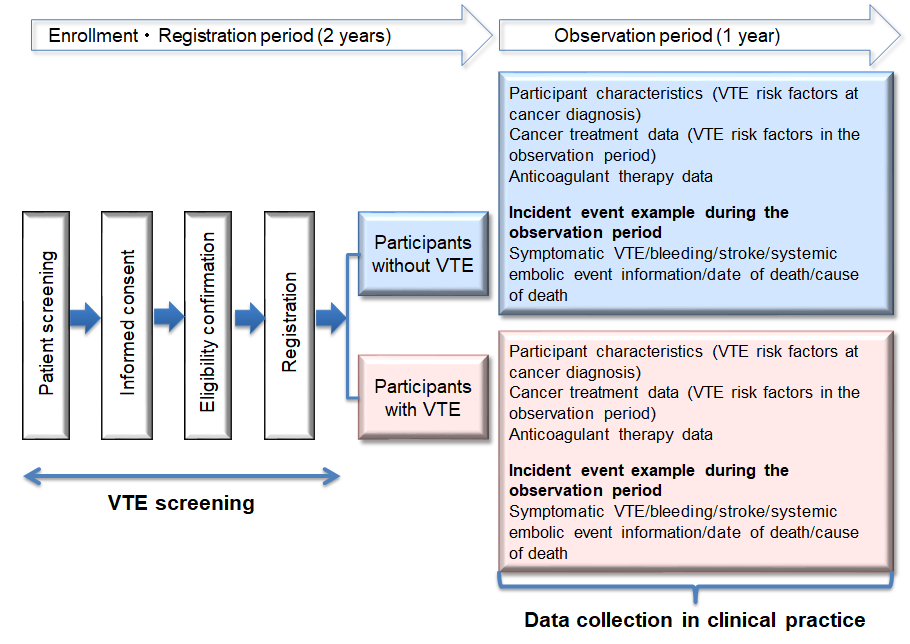


VTE, venous thromboembolism.
