# Supplementary material for: Venous thromboembolism in cancer patients: report of baseline data from the multicentre, prospective Cancer-VTE Registry
Source: Jpn J Clin Oncol. 2020 Jul 27;50(11):1246–53. doi: 10.1093/jjco/hyaa112 (PMC7579341; doi:10.1093/jjco/hyaa112)
Supplement: Cancer-VTE_Registry_Dr_Ohashi_Revised_Supplementary_List_20200605_hyaa112 [file cancer-vte_registry_dr_ohashi_revised_supplementary_list_20200605_hyaa112.doc]

**List of Supplementary files**

One Microsoft Word document file containing the following supplementary data (tables and figures)

Supplementary Table S1. Definitions Used in Venous Thromboembolism Screening

Supplementary Table S2. Prevalence of VTE by Cancer Types and Stages

Supplementary Table S3. Risk Factors Affecting VTE Prevalence (VTE Combined Additive Model [All Data] Including Explanatory Variables)

Supplementary Figure S1. Cancer-VTE Registry Design
